# Public Health Has an Equity Problem: A Latinx's Voice

**DOI:** 10.3389/fpubh.2020.559352

**Published:** 2020-09-11

**Authors:** Jesus Ramirez-Valles

**Affiliations:** Health Equity Institute, San Francisco State University, San Francisco, CA, United States

**Keywords:** academic public health, health equity, Latinos (U.S.), racism and antiracism, schools of public health

## Abstract

Public health has an equity problem. One of the main pillars of our public health system, schools and academic programs of public health, are under the control of white (heterosexual) faculty. They continue to exclude brown, black, and indigenous people from their faculty and leadership ranks. This racism pervades institutional policies and culture and is a major fault in the quest for health equity. In this essay, I center on the experience of Latinx faculty to examine the roots of this inequity and the arguments for diversity and inclusion. I also propose avenues for change and argue for institutional transformation that goes beyond adding people of color to faculty and leadership roles.

Public health has an equity problem. Externally, the field proudly extolls its role in reducing health inequities, here and abroad. Yet, internally, it systematically dismisses people of color from its workforce.

One of the main pillars of our public health system, schools and academic programs of public health, is under the control of white (heterosexual) faculty. They continue to exclude people of color from their faculty and leadership ranks. We, black, brown, and indigenous peoples are not treated equally in academic public health (1).

This is as much irony as it is hypocrisy: our white colleagues are clearly profiting from the appearance of advocating for health equity, while simultaneously keeping faculty of color away. It is racism; and it is a tremendous failure in our strategy to achieve health equity. How can we reduce the burden of disease, illness, and death faced by people of color in this country, when we shut out that same people from faculty and leadership positions tasked with developing solutions? The answer is, of course, we cannot. We cannot be effective when operating from a white, hierarchical, organization, ignoring the talents of people of color.

We cannot allow this exclusion to remain unchallenged; the status quo—in this case—literally kills. It afflicts the most vulnerable communities. And, it is self-perpetuating, because it disenfranchises and demoralizes people of color from pursuing the study of, or profession of, public health, or—once in, forcing a decision to leave academia rather than continue hitting their colored heads up against white walls.

The problem is monumental—it involves policy and culture, from educational pipelines to NIH research funding. Yet, these forces should not stop us. That is why I am taking this step—raising my brown voice, sharing my own experience.

## Exclusion by the Numbers

A snapshot from the current faculty body in schools and programs of public health gives us a sense of the problem. The Association of Schools and Programs of Public Health (2) data show that, nation-wide, we have 2,880 full professors, 189 (6.6%) are Latinx; 2,157 associate professors, 82 (3.8%) Latinx; and 2,335 assistant professors, 8 (0.3%) are Latinx. The total faculty in schools of public health is 9,737 people; 279 are Latinx—that is, 2.9%. We have 62 accredited schools of public health; 84% of them are led by Caucasian deans; 8% by Latinx, 5% by Asian-American, and 3% by African-American. The majority (64%) are men. I am not including Puerto Rico in this count, as the local faculty are not racial minorities in the way we conceptualize racial groups in the (mainland) US context (3).

Race intersects with gender—mostly in a negative way for people of color, but at times, the patterns are vague (4). Heterosexual males are favored and the percent of minority faculty tends to decline with rank. We, faculty of color in public health, experience a “downward trajectory” (1, 4–6).

## The Roots of Inequity

I am the product of Affirmative Action and I fully embrace it and deal with its stigma.

I obtained my MPH and my Ph.D. at the University of Michigan—where I earned scholarships and grants to support my education. In my Master's program cohort of about 30 students, I was the sole Latinx student. The following year, my now dear friend joined the program—she was the only Latina in her cohort. Then, as I stayed in Michigan for my Ph.D., I was the only Latinx not only in my cohort, but throughout all my time there. There were two people of color all that time, a Black woman and I.

Then, I felt different about this than I do now. I thought of myself as Mexican, not as a person of the US—not as a Latino. I had no plans to make the US my home—let alone to embrace my *Latinidad* and internalize my racialized self. Now, 25 years later, I see it differently; clearly. I realize throughout my career I have been the only, the *lonely* brown professor—virtually always the only brown person in academic departments, faculty meetings, search committees, and in promotion and tenure committees. I have felt the bullying and exclusion from my white colleagues.

Looking back, it started when I entered the job market—leaving the protected environment I had created in Ann Arbor. I got several interviews for faculty positions in schools of public health. The praise and encouragement I received came in the form of:

“Oh, nice job, I think all students understood you.” An older white professor told me referring to my accent during my lecture.

“Are you interested in working with farmworkers…? We have had an influx in this area….” Another older white professor told me.

And—my personal favorite— “You would fit well here; it is as corrupt as Mexico.” A Dean told me over our lunch interview.

I ended up taking a job in a school of public health with no Latinx faculty—where the Dean had instructed the chair of the search committee to bring a Latinx professor. My first day, an older white professor welcomed me: “… you finished your dissertation?”

I began my academic career as the only brown face in a faculty of 70. Let that sink in: 1:70. Moreover, 17 years later, I *remained the only* brown person in a faculty of about 115.

At any point in time, there were 4–5 Black colleagues. I became the only minority full professor in the college—one of five Latinx in the entire campus–, and let me come out: the only openly gay faculty member in the college.

Eventually, I became the head of my department. I had the support and mentorship of a senior (white colleague) and an associate dean (a Black woman) who helped me navigate my way to that role. It was not handed to me. I wrestled for it. As department head, I was able to shape the faculty. I hired the second Latinx faculty and recruited four Black professors, among others.

Now, at San Francisco State University, I am part of the 6% of Latinx faculty. In *California*, where Latinx are 39% of the population; in *San Francisco* where we are 15% and in San Francisco State University, a “Hispanic Serving Institution.”

## Racism and Institutional Colorblindness

The problem is clear as it is “color-less.” Why? Why do schools and programs of public health fall so behind in their diversity and inclusion? The answer is simple—racism—unchecked by institutional colorblindness (7).

But, how does racism look like in this context? Two landmark reports in the early 2000s emphatically stated the lack of diversity in the health professions: the Institute of Medicine (8) and the Sullivan Report (9). The latter, unequivocally calls it racism: “Whenever opportunities and resources for faculty appointment, leadership, and research unduly favor a certain racial or ethnic group [whenever] health professions schools impose ethnocentric culture on any other race or ethnic group to that group's detriment” (p. 40). Even more, it concludes: “The historic trend of minority exclusion from faculty, while not necessarily endorsed or advanced by any institutional leader, is kept alive and even reinforced by contemporary institutional frameworks” (p. 41).

## Pipelines Drying Up

The process of exclusion begins early in the toxic, leaky pipeline (6, 10). Latinx dropping out in high school at higher rates than any other group; and while the number of graduates in undergraduate programs has increased (11), we do not see the same in graduate levels and faculty ranks.

Notably, one of the few efforts from the federal government to bring under-represented students in health professions, the Health Careers Opportunities, was brutally defunded by 89% in 2006 (10). Now, 10 states ban race-conscious efforts by universities.

## Self-Fulfilling Prophecies in Recruitment

It then goes to the recruitment and hiring processes overseen by the dominant white culture. Search committees favor white, privileged PhDs, because they look for the traditional standards of success (e.g., number of publications, grants). There is an old unwritten rule in academia; they hire people that look like them. Committees usually do not have mandates on diversity and inclusion—nor do chairs, deans, and provosts.

## Hiring Us Only to Drive Us Out

If we are fortunate and hire a faculty of color, the likelihood that they will stay and flourish is miniscule. The culture is infested with racism and misogyny—and that, crushes us. The lack of diversity on our schools means that a faculty of color is the only one in the department or college. They are perceived as not legitimate scholars (6, 12).

In the eyes of our white, liberal, colleagues, minority faculty might be welcomed, as long as they do not take power away. When I was department head recruiting faculty of color, I was talked about as a “biased brownie.” A white faculty member said to me, as they were advocating for a higher salary, “I am all for increasing diversity, but I deserve a higher salary … I have more experience …” They would go on enumerating every project, publication, and committee they ever worked on.

I have seen senior and junior colleagues struggling under the strain. Faculty of color have come to me crying, looking for a sympathetic shoulder, when they have been called names by students or been hurt by their colleagues. I have seen some leave, walk away from the stress (13).

I have cried too. I have walked into a colleague's office to sob. I have felt my muscles tightening and shaking. I have fought the anxiety and the worrying. I have teared up when a Latina student walked into my office crying about how she was singled out in class. I write and speak this in the hopes of alleviating my own pain.

That is how we have gotten to this point, where faculty of color are invisible in schools of public health—the places proclaiming health for all. I want to make this clear: lack of diversity saves racial and gender bias, and distrust. We must become visible, but in a different way—disrupting the system.

## The Arguments for Inclusion

First, we should demand inclusion and power in school and programs of public health—if for no other reason than our basic civil rights (10). In the face of racial segregation, we demand equal treatment.

Second, it is an argument by the Supreme Court: our education is effective and richer when the classrooms and the textbooks represent all of us. Up until the 1960s, public health students were mostly white, physicians, and nurses (14). Nowadays, students are younger, from a variety of academic backgrounds, and more diverse. Yet, this has not translated into changes in the way we train them. Our education will be much better with black and brown professors. Our students need to see us, people like them.

My third reason is the actual work of reducing health inequities. Our blueprint, CDC's Healthy People 2020, includes public health infrastructure as a goal: “requires health professionals who are competent in cross-cutting and technical skills … and public health organizations with the capacity to assess and respond to community health needs” (15). Effective organizations and strategies require a diverse workforce. A white public health force is not capable of addressing this goal.

## Disrupting Public Health

We cannot allow white supremacy (and its meritocracy) to define what health equity is. It is putting the proverbial “fox in charge of the hen house.” We cannot allow them to profit from our health inequities. Our duty is to disrupt the public health infrastructure, not just change its color.

Conservative forces are dividing and attacking us, in speech and actual corporeal force. Tenure-track faculty are waning around the country (5, 16), and even more in schools of public health. The increase in adjuncts has created fear and conformism, emboldening the ivory tower. Faculty do not want to (and frequently cannot) take risks. We please superiors, reviewers, and founders, not the public, not our spirit. Our work hardly speaks to the daily, current lives and struggles of people.

It is hard to make even small changes. This, however, should not silence us. I, at the very least, will speak up and share what I have learned and what I see as our possible paths to decolonize public health.

At the broader civil rights and policy level, we are talking about reparations for slavery and racism. We can imagine infusing our educational system with resources and truly ensure everybody has access to the best education society can afford.

We have affirmative action policies; they must be used in a holistic perspective for admission process. The goal here, as defined by the Supreme Court, is to achieve a critical mass. They are not perfect, but they do help.

There are a couple of federal programs that provide financial resources, such the Health Careers Opportunities Programs and the Loan Repayment. They are not enough. At our local and immediate context, we must come together and fortify our activism. My five-point action plan for the here and now:

1. **Apply Pressure**. We, faculty of color and our allies, need to unite and pressure our institutions—from board of trustees, presidents, provosts and deans to commit with actions and resources to stop racial exclusionary practices. The Sullivan report (9) made it clear—the directive has to come from top to the bottom. Let us start by reaching out to the faculty of color next to us—or to our allies.
2. **Hold Schools Accountable**. Responsibility for racial equality and health equity go beyond empty declarations of diversity and inclusion. Who is holding our schools accountable for diversity? Accrediting bodies and funders need to do this. Schools cannot do it themselves. Let us ask accrediting bodies and funders to set criteria for inclusion and diversity.
3. **Keep Score**. We should apply a “diversity and inclusion score” in the schools ranking systems (4).
4. **Create Transparency**. Require schools to make publicly available the composition of their faculty and their policies to guarantee ethnic and racial inclusion (4). Getting data on faculty in public health is hard. Not all academic programs are members of ASPPH (which charges expensive fees), and not all ASPPH members report faculty composition details (e.g., compensation, tenure, level of effort). What is more, the data are not publicly available.
5. **Speak up**. Share your thoughts on social media, right now.

Inclusion must come with disruption; if not, this is cooptation. Having colored bodies in faculty and leadership ranks should lead us to a different way of academic work. This requires changing how we recruit and promote faculty and transforming the student body and the curriculum.

## Unite and Disrupt

Public health has a moral conundrum (17). It proclaims to work toward health equity, yet, internally, our schools of public health are profoundly inequitable.

CDC is working on the 2030 Healthy People goals. Achieving health equity is still one of the top aspirations. This is the time to speak up.

Let us stop the “cultural competence” and “diversity and inclusion” jargon (13). This is racism. We must call it out. We must make the white system *include us*. We must demand white liberals to “walk the talk” and decisively value us—people of color—in the ranks and the decision making.

Yet, it is about us not falling into the trap. As with any movement to change an unjust system, it is about uniting in order to disrupt the system that creates these health inequities. We will not decolonize public health only by naming it and debunking the “diversity and inclusion” rhetoric.

Disruption is our public health intervention.

## Data Availability Statement

The original contributions presented in the study are included in the article/supplementary material, further inquiries can be directed to the corresponding author.

## Author Contributions

The author confirms being the sole contributor of this work and has approved it for publication.

## Conflict of Interest

The author declares that the research was conducted in the absence of any commercial or financial relationships that could be construed as a potential conflict of interest.
